# Remarks on Dysentery, as It Prevailed in East Bloomfield, N. Y., during the Autumn of 1848, Being the Substance of an Address Delivered before the Ontario Co. Medical Society

**Published:** 1848-12

**Authors:** T. G. Meachem

**Affiliations:** East Bloomfield, Ontario Co., N. Y.


// BUFFALO MEDICAL JOURNAL

AND

MONTHLY REVIEW.

VOL. 4. DECEMBER, 1848. NO. 7.

### ORIGINAL COMMUNICATIONS.

ART. I.—*Remarks on Dysentery, as it Prevailed in East Bloomfield, N. Y., during the Autumn of* 1848, *being the substance of an Address delivered before the Ontario Co. Medical Society.* By T. G. MEACHEM, M. D. (Communicated for the Buffalo Medical Journal by request of the Society.)

It is a curious and interesting fact, confirmed by the concurrent testimony of medical observers from the age of Hippocrates to the present day, that the human constitution is prone to take on diseased action in different sets of organs or functions, at various periods of the year; or, in other words, that the different seasons of the year are marked by an aptitude to certain determinate diseases in the human system generally. What hoary experience has thus irrefutably established, unaided reason might almost have taught *a priori*, and so have forestalled experience, instead of merely corroborating it. For when we consider the numerous, great, and sudden meteoric changes of the proximate seasons, and the very opposite, though distant conditions of the remote or extreme seasons, together with the multiplied corresponding diversities in the vast vegetable kingdom, with which we hold so intimate a connexion and relation, the conclusion is pressed upon us, irresistibly, that the sensitive and wonderfully complicated organization of man cannot possibly exist in the midst of these ever-varying circumstances, uninfluenced and unaffected, but that, either for weal or woe, it must be powerfully under their control. It is true, the human constitution is so wisely framed, its adjusting and compensating powers are so nicely and accurately arranged, and its recuperative principle is so vigilant, active, and tenacious, that it is capable of frequent and protracted exposure to these changes with little or no detriment, especially where a sufficient knowledge of the laws of life, or physiology, is possessed, to anticipate, and so ward off their evil impressions, or, having felt them, to remove them before they have time to become serious. In many cases, however, the powers of endurance belonging to the system, and the strength of its conservative principle, are so inherently weak, that there is an utter inability to cope with any but the slightest opposing influences; while, on the other hand, a profound ignorance of the physical laws of our nature, or a knowing and wilful disregard or perversion of them, at the same time that it renders that weakness complete imbecility, makes even the finest constitutions the prey, instead of the successful antagonist of these changes of season, particularly where the agencies they develop, are more than usually nocuous and virulent. The individuals, then, who are not included in one or more of these categories, and therefore susceptible to the deleterious influences peculiar to different seasons, are very few; and the only reason why more do not actually suffer than observation proves to be the case, is not because they are not liable to suffer, but, because, fortunately, they are not brought into the immediate sphere of the developing or exciting causes.

Owing undoubtedly to the nature and degree of their changes, the same seasons in different years, in similar latitudes, vary widely from one another in the form, type, severity, and mortality of their maladies, as well as in the age, and, occasionally, the sex of those whom they select for their victims. While some, on the one hand, are marked by nothing inordinate in these respects, exciting no disease but such as is readily amenable to medical treatment, or as mild, even, as to pass by almost unnoticed; others, on the other hand, are marked by the most appalling traits, generating complaints that bid defiance to the best directed skill of the most enlightened physicians. Providentially removed from the sweltering heat and resulting pestiferous influences of the southern climes, to which, *par excellence*, the latter remark applies, and proportionably free from those great national curses, as they have been designated, large cities, which congregated poverty and vice too often convert into vast pest houses, it has been the lot of this portion of the country to enjoy a comparative immunity from disease. Here and there, however, at distant periods of time, the otherwise steady current of health has been rudely interrupted; and, as if to take vengeance for past exemption, sickness and death have run riot in our midst. Such was the case in the Typhoid Pneumonia of 1812, 13 and 14, the Epidemic Dysentery of 1828, the remembrance of both which is doubtless fresh in the minds of veteran practitioners; the Erysipelas of 1845, 6, and 7, and, in some measure, the Diarrhœa and Dysentery of the past autumn. No one at all observant of the state of health in Western New York during that season, could have failed to notice the unusual prevalence, severity, and fatality of our accustomed fall complaints. In some favored localities nothing but sporadic cases of abdominal affections occurred, as heretofore, and they, for the most part, were mild in their form, and brief in their course. But the great majority of places, if I mistake not, suffered far beyond the inflictions of antecedent seasons; while some few witnessed the very maximum of fatality consequent on the direst forms of disease that ever visited our country. As instances of the last class, *many* of our large towns might be mentioned; * and. as a notable instance, out of the state, reference may be made to Lowell. The aggregate number of deaths which occurred in these places, I know not, although I made enquiry to that end, neither, indeed, the number individually; but the public prints emanating from them during the sickly period, bear ample record of the immense mortality. As an example of the wholesale mortality in the practice of one physician alone, in a neighboring city, I was advised through Dr. ———, that, to his knowledge, a brother practitioner lost fifteen cases in a few days. In Lowell no less than sixty died in a day—a number, for the population of the place, that may challenge competition with that scourge of the human race, the cholera, in the very height of its destructiveness. Although our own immediate section of the state happily escaped the dreadful fatality that befell some other portions of it, we by no means passed through unscathed, as the experience of many among us can abundantly testify. In this town, I believe, as well as in Richmond, West Bloomfield, East Bloomfield, Victor, &c., the fall affections were unusually rife, and far more than commonly fatal, though not remarkably so. Such was the case, too, in Livonia, Lima, Geneseo, Nunda, and other towns in the adjoining county of Livingston.

As a general rule, here and elsewhere, the appearance of diarrhœa and dysentery in the late summer and early fall months, has been to some extent preceded by cases of cholera infantum. In many situations this was the case during the past year; in others, not. In East Bloomfield, for instance, I believe there was not a single case of infant cholera, although

* Among them, I have been informed, Syracuse, Lyons, Mt. Morris and Rochester, stand prominent.

there were numerous cases of the other affections. In Rochester, owing, no doubt, to the circumstance of its being a city to which, like all other cities, more or less, there is a constant influx of pauper emigration to increase the many causes of the complaint previously existing, cholera infantum showed itself at an early date, and as soon as the month of June, put on, too, dysenteric symptoms of a well marked character—a complication, in itself, of course, by no means rare, but worthy of comment from its occurring at so early a period, and spreading so widely as it did. In July, the fevers of a typhoid type which visited the city, were noticed to possess an unusual tendency to looseness and irritability of the bowels, and dysenteric symptoms generally, such as occasional mucous stools streaked with blood; and this was especially true with reference to foreigners and the poorer class of persons. About the last of July, dysentery was met with in its decided form, and from that time forward, till the latter part of October, it raged extensively in the city and its environs. In this town, I have been told, the tendency to abdominal disease manifested itself in the form of diarrhœa, or diarrhœa complicated with dysentery, constituting dysenteric diarrhœa; there having been but few cases of genuine dysentery. Up to the 10th of August, there was but *one* case of dysentery in East Bloomfield, of which I am cognizant, and but very few of diarrhœa; but from that date, up to the 10th of November, we had numerous cases of both affections, single and combined. With three or four exceptions at Bloomfield Centre, all but one of a light character, and one or two south and south-east of the Centre a mile or so, the sickness was limited to the course of the stream on which Shepherd's Mills are erected, and its immediate vicinity, together with the neighborhood of a rivulet emptying into this stream not far from the Mills. The portion of the town thus marked out by the sickness, may be represented by a line extending east and west about two miles, following the channel of the creek, with one departing from its centre, south, and reaching about three quarters of a mile. The land through which the creek and rivulet both run, is very low compared with other parts of the town, having a porous and dank soil, and being, in fact, the temporary reservoir of the water which flows from the bordering highlands. Indeed, a large tract of the land crossed by the former stream spoken of, is still an undrained marsh. Whether the fact of these diseases evincing a preference, the past year, to low, moist situations, is a general, or a local one only, I have not sufficient data at hand positively to determine; but if the situations of Lyons, Syracuse, Mt. Morris, Nunda, Rochester, &c., &c., all lying on the line of canal, be allowed any bearing on the question, its general character would seem to be pretty strongly corroborated. Those portions of West Bloomfield and Livonia which suffered most, were likewise low.

The type of the cases, both of dysenteric-diarrhœa and dysentery, which occurred in my own practice, and in that of neighboring physicians, was chiefly typhoid of a well marked, and, sometimes, most malignant character. Many cases which did not evince this type in their incipiency, did so in their subsequent progress, being at first more akin to synocha than typhoid; most, however, manifested the unmistakable symptoms of the adynamic or asthenic state from their outset. A highly inflammatory or sthenic state of the system was so rare, that I do not remember belng called to see but one case of it, and that was in the person of a robust, vigorous young man residing south-east of the Centre, out of the "infected district," though in rather a low and wet locality. As in other seasons, all classes and ages, as well as both sexes, were taken down with the disease. Between the sexes, numerically, I do not know that any preference was manifested, both suffered alike; but the condition of the patient as to the comforts of life, and his age, particularly, made a plain and sad difference in the inability to resist the disorders, as well as in their severity and result. The few deaths that occurred with us, were all among the *children* of the *poor.* And this was mostly so in other places, as Rochester, for example. The majority of the fifteen fatal cases (to which allusion has been made) in a neighboring city, were of the Dutch and Irish emigrants. That poverty and infancy should predispose to the reception of disease, and after its contraction, render it less able to cope successfully with it, is so generally conceded as almost to have become a medical axiom, a "fixed fact." For it is at once apparent, that as the capability of the system to defend itself against the onset of disease, is co-ordinate with the vital force which it possesses; and that as the degree of marked action in a given instance, is by no means commensurate with the vital force, but often runs far beyond a just proportion; weakness and frailty of that force—the legitimate fruits of infancy and poverty—must sooner succumb than strength and power.

The symptoms accompanying the affections of which I propose to treat, were, of course, somewhat varied by circumstances, as age, temperament, habits, &c,, of the patient. In nearly all instances they were gradual in their commencement and progress, following a slow, though undefined course; and were widely diverse from the symptoms attending those rapid species of the complaints which we often meet with, and which are usually associated with an exalted state of the vascular and nervous systems, as denoted by the full, hard pulse, intense pain, &c. A morbid state of the colon and rectum, and other dysenteric symptoms, were prominent traits generally. Genuine diarrhœa, therefore, was far from common; and if certain cases appeared wholly free from admixture with the dysenteric symptoms at their onset, they soon lost their distinctive character for the most part, and merged into union or amalgamation with the other affection. Some cases of this description were made so, I have no doubt, by the use of unseasonable and injurious domestic remedies. In the initial stage of the complicated, and throughout in the unmixed diarrhœa, fever was absent in the majority of instances, and the pulse, skin, urinary secretion, tongue, and appetite, were very often nearly or quite normal; the only indication of derangement in the system, being the frequent and loose stools, together with some pain or uneasiness in the abdomen, felt, principally, just previous to an evacuation. The frequency of this, or in other words, the rarity of severe constitutional disturbance in pure diarrhœa, may be accounted for on the fact, that nearly all the bad cases of diarrhœa were of the complicated form, the simple cases no sooner reaching a certain point, and evincing a proclivity toward severity, than dysenteric symptoms set in, changing at once their character. Dysenteric-diarrhœa, therefore, was seldom a light and unimportant malady, As it occurred under my own observation, it was a combination of the worst forms of diarrhœa with the mildest form of dysentery. Pure dysentery was more aggravated than this, being, as it were, a refinement in morbid action, or farther advancement in it, and reaching nearer that condition of the vital powers which is incompatible with the necessary performance of the various functions of the body, or the continuance of life. Those cases of dysenteric-diarrhœa accompanied with fever from their commencement, were usually ushered in by a sensation of chilliness, or a distinct chill, followed, in a short time, by symptoms of febrile reaction of a moderate grade. To these soon succeeded a feeling of weight or malaise in some portion of the abdomen, centering, after an indefinite period, about the pubic and left inguinal regions, while over these regions there was more or less tenderness on pressure or bodily motion, and, at the same time, there was, not unfrequently, an intolerance of firm pressure over the whole abdominal surface. With this diversity in regard to the period of the febrile accessions, the two divisions of the complaint, the primarily and secondarily complicated, were analagous in their symptoms. The appetite became very capricious, or there was complete anorexia. Nausea was a common attendant, though vomiting, unless from overburdening the stomach by drink, was rare. The tongue was generally, but not in all cases, coated; the fur ou its surface varying greatly in color, thickness, and extent; being sometimes a light, thin, narrow strip only in the centre, or a thin, extended lining of a white color; at other times the lining was thick, and of a brownish cast; and then, again, of a dark hue, dry and cracked into numerous fragments. When not furred, it now and then put on a dry, angry, crisped appearance almost, being very much contracted, and capable of but little motion, so that when asked to show his tongue, the patient could scarcely protrude its point beyond his teeth. As a counterpart to this, there was at times but very little abnormal in the state of the tongue, and that not always in the mildest cases. Some writer has sagely, I think, remarked, that the state of the tongue is a poor index of the degree or extent of inflammation in the large intestines alone, although, generally, a good one in disease of the small intestines and stomach. I think I may safely venture to say, that I have found it true; at any rate, this I have noticed—that I have very rarely, indeed, seen the red, dry, parched appearance of that organ in genuine dysentery, compared with the number of times I have found it associated with inflammatory disease of the intestinal tube higher up. And so, on the contrary, of the opposite condition of the organ. Our last fall's affections disclosed nothing to lead to a renunciation of this opinion. The secretions of the mouth were unnatural and viscid, and bitter to the taste of the invalid. The breath was sour. The alvine evacuations were numerous, thin, and scanty or copious as the one affection or the other, of which the disorder was compounded, predominated. They were very varied in their colour and composition; sometimes consisting chiefly of blood, then of mucus, then of a dark bilious matter, then of mucus mixed with blood, then of a yeast-like substance, and then, again, of a peculiar greenish matter resembling "lumps of flesh stained with bright green paint." The last appearnnce of the stools I witnessed but once or twice; it is produced, according to some, by a mixture of coagulated blood and bile. Tormina and tenesmus were distressing symptoms. The pulse, like the tongue, was differently affected in different individuals, being in some but little influenced by the disease, while in others it was accelerated, quick, tense, and sometimes intermitting. The state of the skin, too, varied greatly, particularly in temperature; for while, in some instances, it indicated a high degree of heat, in others, there was an unnatural absence of it, especially on the extremeties. The low grade of temperature was not, however, generally complained of by the patient, nor was it, indeed, often noticed by him: on the contrary, he still felt as if consumed by a burning fever, to allay the intensity of which he would cast the bed clothes from his body, that it might be exposed to the cooling air. It is said that every thing has its counterpart; and this is but the opposite of that condition of the system induced by malaria, in which, though comfortably warm as tested by the sensation of others, the patient himself complains of insufferable cold. Dysuria was an occasional attendant; and late in the disease, when fatal, floccilatio and subsultus tendinum with coma, supervened. Dysentery being, as I have observed, something like a refinement of morbid action, or a farther advancement in it, its symptoms, of course, were, in the main, those of the disorder just described, with this one wide distinction—that they were of an intenser character, an exaggeration of them. Hence, there was pyrexia, but more severe; the stools were frequent and loose, but less fecal: tormina and tenesmus were present, but they were more importunate and insufferable. In both diseases the disposition to syncope, or exhaustion, was very great, but more so in pure dysentery than in its complication; in fact, in this respect it bore a close and striking resemblance to many of the worst cases of "Epidemic Erysipelas" that have fallen under my notice. The nosological distinctions between the two complaints, were not, however, always strictly observed.

The causes, remote, of these abdominal affections, have already been stated to consist in certain changes or peculiarities of the season. Now, it becomes an interesting, and, to the medical man, pre-eminently an important question, what was the exact agency developed by these changes or peculiarities of season, through the operation of which on the human system, the complaints, so often mentioned, were produced, and what was its *modus operandi?* As medical philosophers have, as yet, failed to discover more than two competent causes for the generation of similar diseases in other places, and in other years, namely, contagion and malaria, we are bound to accept one or the other of them, in the solution of the problem before us, or else acknowledge their combined influence. Let us, then, examine, briefly, the claims which each possesses. (In speaking of the causes, be it remembered, I confine myself, principally, to the town in which I reside.) First, as to the contagion. If by this term we are to understand, what is generally meant by it, the communication, direct or indirect, of a disorder from one who is laboring under it, to one who is not, by contact with an animal poison begotten in the person of the former, I see no valid reason for infering the contagious nature of our late fall affections. Could the disorders be shown to have commenced at a given point, and thence radiated as from a common center, as one writer remarks, the presumption would have been in favor of their contagious nature; but this was in no instance the case. If, again, the circumstances which aid the generation and diffusion of contagion, as excessive filth and vice, crowded and badly ventilated hospitals, &c., &c., had existed here, circumstantial evidence might still have been in its favor; but they did not. Discarding, as we most assuredly must, the idea of positive proof in the premises, the strongest presumptive evidence that can be adduced in support of the position is, that the diseases attacked several persons simultaneously, or at nearly the same time, in the same neighborhood; or that those who went from a healthy locality to nurse the sick, were themselves taken down with the complaints. If I mistake not, the former supposition proves rather the non-contagiousness of the disorders than their contagiousness; for it is at least presumable that a sickness so wide-spread, and so uniform in the period of its appearance, should have had some general and diffusive cause, and could not have travelled by comparatively slow steps from person to person, else would the individuals have been brought down more in succession, more in the order of their contracting the maladies. Moreover, it can be urged as an argument against their general contagiousness at least, that *many* who suffered from them, had, most conclusively, never any communication with those similarly affected. With regard to the second point, it must occur to every one that a removal itself from an uninfected to an infected section of country, was inevitably to endanger the health of the individual so removed, by throwing him into the very midst of the causes, whatever they were, that had already produced the sickness in others. And when to this you add confinement to the sick-room, with its concomitant evils, want of pure air, constant and unrelaxed attention, night watching, mental anxiety, &c., is it at all surprising that these ascribed cases of contagion so often occured? Nay, is it not a matter of wonder that they were not more frequent? What so effectual and unfailing a plan could the ingenuity of man devise to extend and perpetuate disease? It is rushing into the very den of the lion; and strange, indeed, if all, like Androcles, should pass out unhurt. On those who argue for the affirmative of the question before us, devolves the task of defending, on like grounds, the contagiousness of many other disorders which, they will readily concede, are not contagious: such are agues, influenzas, &c. The notion of non-contagion, is, I am well aware, in contrariety to the opinion of older and better men than myself; yet, I candidly, though with some degree of diffidence, assert, that I did not witness *one* instance of reputed communication by contagion, that could not be explained on less questionable, plainer, and more satisfactory principles. After all, I would not wish authoritatively to declare, that, because *I* did not see a case which I was satisfied was the result of contact, and cannot be led by reasoning to the conclusion that such was the fact, others did not witness cases of the kind, and are therefore wandering blindfold in the paths of argument and truth. Positive proof is far superior to negative; and hence, he who can only say, "I have *not* seen," should bow in deference to him who knowingly says, "I *have* seen."

Secondly, what are the claims of malaria? That this subtle and invisible substance has had, at various periods of time, and in different countries, an extensive and powerful influence in the propagation and dissemination of diarrhœa and dysentery, medical history most undeniably shows. The coincidence of these diseases with the great first-fruits of malaria, the ague, or their alternation with it, has been of frequent occurrence for centuries. According to Watson, the decline of diarrhœa and dysentery in the metropolis of England, has been contemporary with the disappearance of ague, and other severe disorders, which, previous to the great fire, were produced by the bad air of pent up streets and filthy sewers. This memorable event, which was considered at the time a national cause, he therefore regards as a great national blessing. Speaking of the former appalling mortality from dysentery in London, he remarks, that "Dr. Heberden, in his valuable work, 'On the increase and decrease of different diseases,' shows that in the seventeenth century the number of deaths set down in the bills of mortality under the titles of bloody flux and griping in the guts, was never less than 1000 annually, and in some years exceeded 4000. For twenty years together, viz:—from 1667 to 1692, they every year amounted to about 2000. During the last century the number gradually dwindled down to 20,000. From the disappearance of the dysentery we may date the extinction of the ague." The same remark holds good with reference to the paludal districts of Holland and other countries. There must, therefore, have been an identity in the causes of the affections. The best explanation of the connection and just relation of the diseases, is that given by the author already referred to, and as his language is luminous and concise, I trust I shall be pardoned for quoting him again. He says:—"repeated paroxysms of ague are attended with extreme and increasing congestion of blood in the internal organs; of which congestion, the tumid spleen is the effect and token. Now whatever gorges the splenic vein, gorges its tributary the inferior mesenteric, which carries the blood from the rectum and descending colon. Upon such congestion of the mucous membrane, inflammation is readily engrafted." Now I know of no reason why the spleen alone should be dwelt on thus emphatically, provided engorgment of any other abdominal organ will bring about the same state of the intestinal mucous membrane. A reference to the etiology of piles and hemorrhage from the bowels, shows us that this is true in relation to the liver. Congestion of that organ, however, though a frequent concomitant or effect of the ague, is not invariably so; for in some parts of the country where malaria prevails, we meet with such derangement, almost daily, in persons who never had the least premonition of the ague. It follows, therefore, that malaria may become the cause of inflammation of the intestinal canal through the medium of the liver, without the antecedence of intermittent fever. With these facts at hand, together with those furnished by the history of the diseases we are considering, as witnessed generally in this vicinity, we have most abundant warrant, I think, for the conclusion, that malaria was the great prime cause of those diseases; producing them indirectly, not through the agency of the spleen as much as of the liver. Indeed, how can the data be tortured to any other deduction? Let us investigate, for a moment, the facts connected with the history of the disorders. First, our autumnal months were characterized by protracted heat and drought, the two prime requisites to the production of malaria. Secondly, the diseases were most rife, and most severe, in and about low and, usually, moist situations. Thirdly, in these localities they appeared first, and remained latest. Fourthly, bilious difficulties of nearly all grades, from the simplest derangement up to bad cases of remittent fever, were quite common, *so* common, indeed, that almost every one labored under them more or less. The bilious countenance met you wherever you turned. More than once was it observed to me by those who had recently come from the west, "you have far more miasm here, than we have; your faces wear more decidedly the sallow token of its existence." Fifthly and last, the ague itself, to some extent, appeared in many of the infected districts; showing, conclusively, the presence of malaria in those districts, and yielding a fair presumption of its existence in other districts near by, and somewhat similarly circumstanced. A few cases of this affection, occurred in East Bloomfield. At Honeoye Flats and South Livonia, both of which places are situated at the foot of a lake, intermittent fever was exceedingly prevalent. My brother, who practices at the latter village, informed me, that there were in its immediate vicinity, at one time, no less than thirty ague-and-fever patients. Subsequently, as at the Flats, diarrhœa and dysentery developed themselves there, attacking, irrespectively, those who had had the ague, and those who had not.*

On a review of these facts, thus briefly enunciated, (for I would not be prolix,) does it not appear evident that the great element in the production of our late fall disorders, was malaria? And is it not equally evident that its *modus operandi* was by causing, sometimes, congestion of the spleen, but, mostly, congestion of the liver, thereby superinducing engorgement of the intestinal mucous membrane? How far these explanations will apply to other sections of the state, and the large towns alluded to, as Rochester &c., I cannot say; but as they were all exposed to the same influences of the season, and were chiefly alike in their situation, I have no doubt they will apply, at least, measurably. In the latter instances, poverty and foreign emigration must have conduced largely to the mortality.

The plans of treatment pursued in the complaints under consideration were various; while the modifications to which those plans were subjected, were numerous, almost, as the individuals to whose hands the treatment was consigned. Paradoxical as it would seem, without taking into view the immense influence of locality in moulding and altering disease, success, to a certain extent, attended all the methods employed; in many instances, on the contrary, they were all strangely and signally inefficient. The principal methods adopted and recommended, may be classed, when analyzed, under the following heads: the depletory, the mercurial, the sedative, the sudorific, the purgative, the astringent and the stimulant. To suit the opinions or theories of the practitioner, every one of these was conjoined with one or more of the others In the cases of simple diarrhœa referred to, much treatment was seldom necessary. Some were promptly relieved by domestic remedies, as a dose of bilious pills, a draught of brandy and loaf sugar, or salt and vinegar, a decoction of smart-weed tea, or oak bark, &c. When called on myself to prescribe, I generally ordered a purgative dose of calomel and castor oil, to be followed by Dover's powder, combined, when there was evidence of much biliary derangement, with equal parts of hydrargyrum cum creta, or small doses of calomel. Where there was an appearance of mucus in the stools, I frequently made use of the powders of ipecac and rhubarb as recommended by the German physicians, and so highly praised by Stokes; and with the success of this plan,

* In further corroboration of the doctrine maintained above, it was stated to the society by Dr. Carr, of Canandaigua, that some western physicians were in the habit of prescribing *quinine* in dysentery as in the ague, and with complete success; viewing the *causes* of the two affections as identical.

as in former seasons, I had ample reason to be satisfied. In two or three instances connected with repletion of the stomach, I, of course, gave an emetic. Dysenteric diarrhœa, and dysentery, being much severer diseases than the simple form of diarrhœa, a more prompt, longer continued, and, where circumstances would permit, more active course was necessarily adopted. Where they were sufficiently dynamic — which was very seldom with us—venesection premised all other remedies; and the guide to the employment of the lancet, was not as much the condition of the pulse, as the degree of tormina and tenesmus complained of by the patient, as well as the bloody character of the evacuations, the amount of fever, thirst, &c. For, as Dewees remarks, "perhaps there are no affections of the system in which the pulse is so uncertain a guide as those of the alimentary canal." Never did I find it necessary or safe, as I thought, to bleed more than once. After V. S., I generally administered a dose of calomel and castor oil, to unload the liver, and evacuate the contents of the bowels; and with this remedy, as with the lancet, I observed great caution, finding for the most part, on experience, what I had been led to infer from previous practice and reflection, that the frequent repetitions of the purgative, instead of relieving or allaying the complaint, tended only to heighten and exasperate it. I know that in making this assertion, I am treading on disputed ground, and giving an opinion on a subject about which, to use the language of Condie, "there is much discrepancy of opinion." Not to break the thread of my remarks, however, I will postpone, for a moment, the mention of my reasons for this conclusion. After the operation of the physic, I put the patient on a powder or pill composed of ipecac, ½ gr. blue mass and extract of hyosciamus, each, one gr., every three hours, and gave him at bed time 10 grs. of Dover's powder. This, it will be perceived, is the method advised by Condie. If the case was severe, and I wished to allay irritation, and produce the effects of mercury promptly, I used, in lieu of the blue mass and henbane, calomel and opium, or morphine. This was continued till amendment took place, or some symptom or condition arose, such as ptyalism, to contra-indicate the further employment of one or other of the articles. The peculiar depressing effects which sometimes follow, secondarily, the administration of opium, I never saw more clearly manifested than in two or three instances of these diseases: so great were they, that although no more than a moderate dose of the narcotic had been taken, the patient appeared on the point of swooning. The cause of this unusual susceptibility may be found, I presume, in the adynamic type of the disorder. After the discharges from the bowels had amended somewhat, I used a combination of ipecac, acetate of lead, and opium, which I found to procure marked relief from the symptoms of termina and tenesmus. As early in the diseases as I dare make use of them, I employed various other astringents combined with opium in some form; doing so much earlier, and plying them more assiduously than I ever found it necessary before. The oil of turpentine and balsam copaiba, too, I prescribed in the same way. Fomentations to the bowels I ordered in nearly all cases. After the acute stage had subsided, I applied blisters to the abdomen, in the commencement of the season, but as they did not seem to afford any compensation for the uneasiness and irritation they occasioned, I almost entirely discontinued them. One case, however, was, I think, essentially benefitted by their repeated application; and that was a case in which, according to Twining, the cœcum was inflamed, as indicated by "pain on pressure of the abdomen over the region of that intestine, and a rounded, doughy, inelastic tumor, both sensible to the hand, and visible." To relieve the tenesmus, the common and highly beneficial remedies of injections of cold water, or opium rubbed up with starch, were given; but very rarely, indeed, did I sanction the use of opiate tinctures in enemas in the early stages, for the reason that I considered them unfit. And candidly, on comparing the pathology of the diseases with the medical properties of proof spirits, does it not look inconsistent to apply to a highly inflamed and exquisitely sensitive membrane, deprived of its natural defensive mucus, so active a stimulant? It may be answered, perhaps, that strong solutions of the nitrate of silver are used in the same manner with the most gratifying success. Granted. But the two articles are not designed to fulfil, precisely, the same indications; the opiate tincture is to produce a sedative effect by suppressing or suspending nervous sensibility; the caustic, to destroy it. Hence the former has no need to be combined with the stimulus of alcohol, which, in fact, becomes a partial neutralizer of its intended action—a stimulus placed in the unhappy medium, being too potent to be innocent, and too feeble to be efficacious. The favorite prescription of Morsely to subdue tenesmus, acetate of lead 1 dr., tepid water 8 oz., I occasionally exhibited. The daily employment of the warm bath in the decline of the disorders, was not forgotten. Overlooking many minor points of treatment, calculated to conduce rather to the comfort and convenience of the patient than the immediate removal of his complaint, this comprises the general medical treatment I learned to be best adapted to my own cases. One indication, however, not mentioned, I found it imperatively necessary to keep in mind; which was the careful husbanding of the patient's strength, and a vigilant guarding against collapse. To this end, mild nutriment, as rice water, and chicken broth, was given at an early date, and, soon after, stimulants and tonics, either singly or combined, in as large quantities as the system could bear. The practice of many physicians differed materially from this. Some of my acquaintances placed their chief reliance on one full bleeding, one thorough operation of a cathartic, and the use of the vapor bath so as to produce copious diaphoresis; following these up by powders of calomel, ipecac, and morphine, coupled with fomentations to the abdomen. By those who put this method of practice in force, the effects of the vapor bath were praised almost immeasurably; to use their own expression, "it was the very sheet-anchor of their hopes." Other practitioners, acknowledging no difference in the type of our late fall complaints and those of previous seasons, classing them promiscuously as sthenic, strenuously urged, and heroically adopted the treatment pursued by them in the past; and thus the very remedy which, of all others, physicians in this part least used, and most feared, to wit, venesection, was by them frequently, fearlessly, and, if we are to credit their assertions, successfully employed. A favorite prescription with some, was, small, repeated doses of calomel and ipecac, with an occasional dose of opium. Some relied mainly on astringents, such as acetate of lead, combined with Dover's powder. Others made *stimulants*, with opium, their principal dependence. A great many trusted to the little sugar pills, and, as a medical friend remarked, died or got well, the best way they could, by the "Vis Medicatrix Naturæ."

A word or two in relation to the question of purgatives in these complaints, which will apply to dysenteric affections generally, and I have done. I have said that this is a vexed question: it is so. For while the great body of American Practitioners is divided on it, it constitutes one of the lines of demarcation between the French and English schools of physicians, the latter contending for, the former against it. Who shall decide where so *many* doctors disagree? It is not for me to assume the station of the umpire, only so far at least as I am myself concerned, and I would not, therefore, without reason, foist my notions of the subject on the consideration of the Society; but the responsibility of the situation which, as members of the Society, we *all* occupy, compels me to state, unhesitatingly, that the plan so highly lauded by some physicians, of giving frequently, in nearly all cases and modifications of dysentery, cathartic doses of calomel, or calomel and castor-oil, or rhubard, or rhubarb and magnesia, &c., &c., I look upon as uncalled for, injurious, and cruel, and therefore decidedly *bad practice.* And for the following reasons: First, because it is diametrically opposite to that cardinal law of both medicine and surgery—derived from the example of nature herself—which requires injured and inflamed parts, to be kept in the most passive and quiescent state, and the necessity for the exercise of their functions to be diminished as much as possible. Witness the use of the bandage in restraining thoracic motion in penetrating wounds of the chest; the complete rest enjoined in sprain of the ancle, or inflammation of the knee-joint; the prohibition of the use of the eye in ophthalmia; the suppression of noise and seclusion of light in phrenitis; the forbiddance of any article making requisition on the digestive powers of the stomach in gastritis, &c.; and that from which, among other examples, the foregoing practices have been imitated, the partial cessation of costal motion in pleurisy and pneumonia, the abdominal muscles acting vicariously. Secondly, because it is directly opposed to another capital law of both medicine and surgery, which forbids the application of irritants to an already irritated surface, except in certain classes and stages of disease, (and then only in the instance of certain medicines,) neither of which exceptions, applies to the question at issue. Thirdly, because the frequent administration of purgatives has often the effect, especially in persons of nervous temperament, and weak digestive organs, to unsettle and nauseate the stomach, and render it incapable of retention, thereby—while the administration of other articles is unduly postponed—through the mysterious yet powerful influence of sympathy, compelling the patient repeatedly to stool, and inducing an unnecessary and hurtful degree of exhaustion and suffering. Fourthly, because experience has pretty satisfactorily proved that those hardened feces termed scybalæ, which were formerly supposed to exist in the bowels in nearly all cases of dysenteric complaints, and to dislodge and evacuate which purgatives originally came into vogue, are found only in a few cases; which paucity can not longer justify the continuance of a general rule. Fifthly, because it savors too much of the Hahnemanic dogma, "similia similibus curanter," which we, as physicians of the old school, repudiate.

For these reasons do I eschew the doctrine of frequent purgation in dysentery.

East Bloomfield, Ontario Co., N. Y., Nov. 1848.

